# Comparison of Local and Systemic Ciprofloxacin Ototoxicity in the Treatment of Chronic Media Otitis

**DOI:** 10.5539/gjhs.v6n7p144

**Published:** 2014-09-18

**Authors:** R. Samarei

**Affiliations:** 1 Head & Neck Surgeon, Urmia University of Medical Science, Urmia, Iran

**Keywords:** ciprofloxacin ototoxicity, chronic media otitis

## Abstract

**Introduction:**

Chronic media otitis is a common cause of reference to ear, nose and throat clinics and the treatment is one of the health problems among ENT specialists. Ciprofloxacin drop that is of fluoroquinolone drug class due to good treatment effect is now widely used in the treatment of chronic media otitis. Due to the widespread use, it seems proper research on the human population has not been taken to ensure its non-toxicity in the inner ear, therefore comparison of local ciprofloxacin ototoxicity with systemic in chronic media otitis is investigated in this study.

**Materials and Methods:**

This study was conducted as a randomized clinical trial. Prospective methods were considered and the number of samples in the study group was 40 patients that were treated with ciprofloxacin drops. And in the control group 32 patients with chronic media otitis who were treated with ciprofloxacin tablets. The collected data was analyzed using SPSS software.

**Results:**

Statistical indicators of different frequencies in air conduction (AC) in both groups showed, there was significant improvement in hearing thresholds at frequencies of 250, 8000, 1000 in air conduction for the group receiving drops compared to the group receiving tablet. Based on statistical indicators in different frequencies of bone conduction in the two treated groups, there was significant difference in the two groups receiving tablets and drops only at a frequency of 4000Hz that drop impact improves hearing threshold and in contrast in the group receiving tablet hearing loss was seen in the frequency of 4000.

**Discussion:**

Topical ciprofloxacin is a safe and uncomplicated ototoxic drug that is an effective antibiotic used in the treatment of refractory chronic otitis those dregs such as pseudomonas aerogenusa and staphylococci resistant to methicillin are responsible for it, which in the usual doses has not harmful effects on hearing hairy cells.

## 1. Introduction

Ciprofloxacin drop that is of fluoroquinolone drug class due to good treatment effect is now widely used in the treatment of chronic media otitis. Due to the widespread use, it seems proper research on the human population has not been taken to ensure its non-toxicity in the inner ear, therefore comparison of local ciprofloxacin ototoxicity with systemic in chronic media otitis is investigated in this study in order to achieve the following objectives (Jang, 2014). This study was conducted to determine whether the ciprofloxacin drop that is already used widely as a relatively new treatment in patients with chronic media otitis instead of other topical medications such as gentamicin has ototoxicity effects or not (Xia, Kong, & Wang, 2014). Ototoxicity of drugs such as chloramphenicol, neomycin and gentamicin has been proven over the long term (Cumming charls et al., 1998). Ciprofloxacin drop is used in association with active infection as a substitute for appropriate coverage of the dregs causing chronic infection in the middle ear. Few investigations have conducted on quinolones’ ototoxicity such as ciprofloxacin as ear drops and it is noteworthy that this research has been conducted on laboratory animals as iatrogenic that their tympanic membrane was ruptured (Darrj, 1994).

Considering the widespread use of ciprofloxacin drops in patients with chronic media otitis and due to the presence of active ear, nose and throat center, an equipped and precision audiometry clinic with experienced staff in Loghman Hakim Hospital, we are to determine the effects of drug ototoxicity. In the study comparing audiograms before and after taking ciprofloxacin ear drops is determined whether or not topical drugs have adverse effects on hearing. Patients with chronic media otitis that are treated with ciprofloxacin considered as control group. Chronic media otitis is a common cause of reference to ear, nose and throat clinics and the treatment is one of the health problems among ENT specialists. In a long period because of the hearing loss and the presence of infectious foci in the body and serious complications and even deathful due to the disease, the person stricken with disabilities, thus the basic treatment is necessary (Cumming charls et al., 1998). The ultimate treatment of chronic media otitis is eradicate infection surgery and ultimately reconstructive surgery of the middle ear, but to prepare the ear before surgery, dry ear and non-active infection for at least three months is necessary for successful operation (Cumming charls et al., 1998). Furthermore, where there is no possibility of surgery and removing the root of disease due to bad physical conditions (age, underlying diseases, etc.), there is a need for safer antibiotics and preferably local for repeated periods. This is because the use of an antibiotic with low and acceptable ototoxicity is needed for a long time that cause ototoxicity in the cochlear system and the hearing loss is not improved (Cumming charls et al., 1998). Based on previous research, neomycin and gentamicin ear drops were containing ototoxicity at some levels, studies also have been conducted on a relatively new drug class known as the fluoroquinolones, which are antibiotics with proper effect on responsible dregs in chronic media otitis that in this group, ciprofloxacin and ofloxacin are the most studied drugs, in animal models a high volume of research has been conducted and hearing examination has been performed based on ABR and histopathology of auditory cells after drug use that the results are shown the non-toxic nature of the fluoroquinolone drug group (Russell et al., 2001; Ozagar et al., 1997; Dohar et al., 1998; İkiz et al., 1998). Now quinolones in their leadership ciprofloxacin with notion of the lack of ototoxicity and proper response to therapy is used as an alternative to aminoglycosides. This review will determine whether we can use ciprofloxacin to treat ear infections safely in the extended periods without fear of ototoxicity or not?

## 2. Methods

This study was conducted as a randomized clinical trial. Methods are considered prospective and observational and analytical techniques for search results and overall objective was to compare the ototoxicity value of systemic and local ciprofloxacin in the treatment of chronic media otitis, for this purpose the patients should be recognized with chronic media otitis that these patients have purulent active otorrhea and perforated tympanic membrane for more than three months (independent of quality ratings based on observation). Investigated population was patients referred to ear, nose and throat clinic of Loghman Hakim Hospital with chronic media otitis clinical criteria. Sampling was random and continuous and those with the following factors were excluded.

1) Age less than 18 years
2) Pregnancy
3) Breastfeeding
4) Recent local drug
5) Proposals for viral infections
6) Sensitivity to the fluoroquinolone drug group
7) Use of systemic medications that are ototoxic.
8) Concurrent infection of the middle and external fungal

40 patients in the study group were treated with ciprofloxacin drops. And in the control group 32 patients with chronic media otitis, who were treated with ciprofloxacin tablets. After the legal procedures and obtaining the necessary permits for the project, all patients with a history of tympanic membrane perforation that had purulent otorrhea for three months or more underwent examination and regular suction for outer middle ear washings. After suction and initial evaluation of the patient in terms of qualification to entrance into the plan, after the readiness of patients, Form 1 was completed. After completing Form 1 patient were referred for hearing evaluation to audiometry center. Coordination in conjunction with audiometry at high frequencies (frequencies above 8000) for the cases was conducted by co-design in the audiometry center. Ciprofloxacin drop was given for some of these patients and for a number ciprofloxacin was given orally. The drugs were administered for 10 days for a group of patients who were using eye drops, drops 2 times a day, each time 2 drops in the affected ear and for patients who were in the control group was given ciprofloxacin tablet 500mg twice daily. How to properly use ear drops were explained to the patient and advised against the simultaneous use of the drug to the patient during this period. Complete description of the possible side effects such as dizziness, hearing loss and tinnitus, or a feeling of heaviness in the ear was given to patients to remind them that if any of the symptoms listed above detected refer to the clinic for evaluation.

Patients were seen on the tenth day. In case of continued discharge and lack of above-mentioned side effects, the medication was continued for another week and in cases where there was clinical response at the tenth day treatment is stopped and was advised to keep the ear dry. 20 days until the next visit, a re-examination of the ears was taken at that time. In the absence of otorrhea information Form 3 was completed for patients. And patients were referred to audiometry center for audiometry and completion of Form 4. Patients who did not have any good response to ciprofloxacin drops, another method of treatment received and was dropped from the plan. Also, patients who have complications of drug use, patients who were added to the hospital during the treatment of fungal otitis were excluded from the study. The collected data were analyzed using SPSS software (Dalfard & Ranjbar, 2012).

## 3. Results

Audiometry of patients that were included in the plan was divided into two groups of 40 cases and 32 controls. For each person in a different frequency bone conduction and air conduction was found for the first day and thirtieth day and the difference of the threshold of hearing audiometry of the first day and thirtieth day for bone conduction at frequencies of 250, 500, 1000, 2000, 4000, and for air conduction at frequencies of 250, 500, 1000, 2000, 4000, 8000, 10000, 12000 were calculated. T-Test was used for statistical analysis of the results that Table 1 is related to statistical indicators in air conduction and Table 2 is related to the statistical indicators in bone conduction that were obtained in two groups.

**Table 1:** Statistical parameters of different frequencies in air conduction (AC) in the two treated groups

DRUG		N	Mean	Std. Deviation	Std.Error Mean
AC250	Drop Table	40 32	+6.8750 -0.9375	12.0728 6.8906	1.9089 1.2181

AC500	Drop Table	40 32	-0.1250 +1.0938	10.5300 6.6883	1.6649 1.1823

AC1000	Drop Table	40 32	-1.3750 +1.0938	8.2421 9.6499	1.3032 1.7059

AC2000	Drop Table	40 32	+3.5000 +4.6875	11.0477 6.3421	1.7468 1.11211

AC4000	Drop Table	40 32	+2.2500 +1.8750	11.091 7.4865	1.7537 1.3234

AC8000	Drop Table	40 32	+5.5000 -1.2500	14.4939 10.8509	2.2913 1.9182

AC10000	Drop Table	40 32	+6.2500 +0.9375	12.4936 6.7725	1.9754 1.1972

AC12000	Drop Table	40 32	+4.2500 +3.9063	9.9066 5.3483	1.5664 0.9454

Based on this table the improvement of hearing thresholds at frequencies of 250, 8000, 1000, in air conduction for the group receiving drops is significant compared to the group receiving oral intake.

**250 T=0.325 Significance 0.02 p<0.05 frequency**

**8000 T= 2.188 Significance 0.032 p<0.05 frequency**

**10000 T-2.163 Significance 0.034 p<0.05 frequency**

In other frequencies symptoms for the sake of hearing loss was not seen in both groups significantly.

**Table 2:** Statistical parameters of bone conduction at different frequencies in the two treated groups

DRUG		N	Mean	Std. Deviation	Std.Error Mean
BC250	Drop Table	40 32	+2.3750 +1.8750	4.0805 6.0575	0.6452 1.0708

B500	Drop Table	40 32	+3.000 2.0313	6.0764 6.00721	0.9608 1.0734

BC1000	Drop Table	40 32	+0.3750 +1.0938	7.1061 6.0554	1.1236 1.0705

BC2000	Drop Table	40 32	+1.6250 +3.2513	4.7214 8.900	0.8771 2.1591

BC4000	Drop Table	40 32	5.5470 12.2134	0.7465 7.4655	+2.5000 -4.8438

Based on these statistical parameters significant difference in the two groups receiving pills and drops was only at 4000Hz frequency that drop impact was on improvement of hearing thresholds and unlike in the group receiving tablet hearing loss at 4000Hz frequency was seen.

4000 T=3.393 Significance 0.01 p<0.05 frequency.

## 4. Discussion

Ototoxicity of topical ciprofloxacin compared with other medications was performed to ensure non-ototoxicity on hearing hairy cells toward other topical antibiotic drops, which is known toxic, according to this new medication on laboratory animals that in these animals in most cases caused as iatrogenic perforation of the tympanic membrane and previously had no history of middle ear disease as chronic otitis. In most of these methods it is used to measure the impact on the auditory brainstem (ABR) and in a few cases morphology of hearing hairy cells (effect on survival and cell death, and length of the hearing cells). Therefore, for some aspects of these studies can be imported objections.

**A)** In previous studies laboratory animals had not chronic media otitis (mucosal changes). Therefore, the effect of the drug with the patient’s condition that suffers from active otorrhea with chronic middle ear changes has two major differences.

1) In terms of high drug absorption from anatomical tracts between the middle ear and the inner ear in non-otitis ears.

2) Unable to assess hearing in situations that mucosal inflammation and mucosal changes are treated with topical form becomes below or disappears. Because studies were in animals that have no middle ear pathology except the study of Mr. Dohan that was on laboratory animals of prior research, media otitis was created with pseudomonas aeruginosa. But in our investigation all patients had otorrhea infection and chronic media otitis (Dohar et al., 1998).

**B)** Most of the studies were based on the hearing control of ABR that threshold with ABR did not commonly measure with high accuracy as conventional audiometry and high audiometry frequencies. Also the status of conductive and sensorineural cannot be surveyed seperately. But because the cases were animals, there was not the possibility of PTA audiometry. In Mr. Brownlee’s research also based on measuring ciprofloxacin hearing thresholds there was only a slight increase in hearing threshold at both frequencies 4KHz, 8KHz that showed a relative equality with the results of our study.

**C)** Another criticism of previous studies showed the inability of ototoxicity of topical antibiotic drugs that were formerly known as toxic that is even in human study by Azgar et al. between gentamicin and ciprofloxacin none of them was toxic for patients, of course this can be due to the small sample population range (Ozagar et al., 1997).

In our study, 72 patients were studied in two groups. In the case group 40 patients with chronic media otitis that treated with ciprofloxacin drop based on conducted audiometry in the first and thirtieth day and statistical analysis of the differences between the audiograms any ototoxicity at frequencies of 250, 500, 1000, 2000, and 4000 occurred in bone conduction for patients. In air conduction also significant loss in the level of hearing at frequencies from 250 to 12,000 was not observed but there was significant difference between improvement of hearing threshold compared with control group at frequencies 250, 8000, 10000 (p <0.05). In the group receiving ciprofloxacin tablets increase hearing thresholds in bone conduction were observed at a frequency of 4000. Because of this difference was significant between the two groups receiving pills and drops (P<0.05). Note that there was not the possibility of the review of hearing threshold at frequencies above 4000 by air conduction, through generalized bone conduction equation in high frequencies, that is more than 8,000 was used indirectly. It is noteworthy that the improvement in hearing threshold in air conduction at frequencies above 8000 represents lack of increase in sensory and neural hearing thresholds in both groups receiving pills and drops at high frequencies, which conclusion is obtained in favor of non-toxicity of the ciprofloxacin drops in ears with chronic media otitis that usual doses prescribed for them. With regard to previous studies and this study can be found the fact that topical ciprofloxacin is used as an effective antibiotic in the treatment of chronic refractory otitis that dregs such as pseudomonas aeruginosa and staphylococcus resistant to methicillin was responsible for, are safe and uncomplicated ototoxic drugs that the usual doses prescribed have not harmful effects on hairy hearing cells. Also, because enters directly into the position of the infection the systemic form of the drug due to scars of sub-epithelial also creates less vascular tissue caused by chronic mucosal changes has not the ability to achieve effective levels in infectious position (middle ear) and in reducing mucosal inflammation caused by active infection due to directly reach the inflamed tissue is preferred to the type of systemic ciprofloxacin.

## 5. Suggestions

1) To increase the statistical accuracy and definite results the high population of the study as a principle must be considered
2) To raise the standard of drug to ensure the testing accuracy and compounds before the implementation of this plan should ensure that an Iranian company manufacturing medical drugs is used.
However, in the discussion of the external validity of these results cannot have a confidence in their generalization to the abroad cases, because definitely the quality of the drug is different in abroad, and its outcome is also different.
3) To ensure the use of drugs and the accuracy of the results, it is better that the medicine is given by skilled and experienced people to the patient that probably the incorrect use and any impairment dosage should not lead to a distortion of the results of the research that of course, this action is not possible in the community of the research. This should be considered as elimination of the correct usage factor of medication in future research.
